# Ultra-Processed Food Intake Is Not Associated with Systemic Inflammation in People with HIV

**DOI:** 10.3390/nu18081211

**Published:** 2026-04-11

**Authors:** Ziad Koberssy, Aaron A. Fletcher, Joviane Daher, Jennifer E. Murphy, Jhony Baissary, Ornina Atieh, Kate Ailstock, Morgan Cummings, Nicholas T. Funderburg, Grace A. McComsey

**Affiliations:** 1 School of Medicine, Case Western Reserve University, Cleveland, OH 44106, USA; ziad.koberssy@case.edu (Z.K.);; 2 Clinical Research Center, University Hospitals Cleveland Medical Center, Cleveland, OH 44106, USA; 3 Division of Medical Laboratory Science, School of Health and Rehabilitation Sciences, The Ohio State University, Columbus, OH 43210, USA

**Keywords:** people with HIV (PWH), ultra-processed foods (UPF), NOVA classification, systemic inflammation, cardiometabolic risk, body composition, diet

## Abstract

**Background/Objectives**: People with HIV (PWH) remain at high risk for cardiovascular and metabolic complications despite effective antiretroviral therapy (ART). Diet quality is an important modifiable factor that may influence these complications. Diets high in ultra-processed foods (UPF) have been linked to adverse metabolic and inflammatory profiles in the general population, but their impact on PWH remains poorly understood. The NOVA 4 classification categorizes foods by degree of processing, from unprocessed/minimally processed (NOVA 1) to UPF (NOVA 4). **Methods**: We conducted a cross-sectional study of adults with virologically suppressed HIV on stable ART. Assessments included dietary intake consisting of 24 h recalls analyzed with Nutrition Data System for Research software (NDSR) and classified into NOVA categories by a registered dietitian and the following characteristics: body composition (total and regional fat by DEXA and CT scan abdomen), cardiometabolic variables (glucose, HbA1C, HOMA-IR, lipids, blood pressure), and biomarkers of inflammation, immune activation, and gut integrity quantified by ELISA. Patients were stratified into NOVA 4 groups based on the median and quartile proportions of total energy intake from NOVA 4 foods. Associations between dietary NOVA and outcomes were analyzed using generalized additive models (GAMs) adjusted for age, sex, race, and CD4 count. **Results**: Among 222 PWH (mean age 45.4 ± 14.2 years; 31% female; 66% non-white; BMI 30.61 ± 7.91 kg/m^2^), median NOVA 4 intake was 45.6% of total energy intake. Participants with higher vs. lower NOVA 4 intake showed differences in diet quality, but in GAMs, higher NOVA 4 intake was not associated with higher levels of inflammatory, cardiometabolic, gut integrity, and body composition variables. **Conclusions**: In PWH, UPF consumption was high but not associated with markers of cardiometabolic health, systemic inflammation, or gut integrity. This may reflect the multifactorial nature of the heightened inflammation in PWH, potentially obscuring the effect of diet.

## 1. Introduction

People living with Human Immunodeficiency Virus (HIV) (PWH) are experiencing increased longevity due to widespread access to modern, effective antiretroviral therapy (ART). However, this improvement in life expectancy has been accompanied by a persistently elevated burden of cardiovascular disease (CVD) compared with the general population. The mechanisms underlying this excess risk are multifactorial and include traditional cardiovascular risk factors, chronic immune activation and systemic inflammation, adverse metabolic effects of ART, and broader social and structural determinants of health [1,2]. Persistent inflammation, even in the setting of virologic suppression, is recognized as a central driver of cardiometabolic morbidity in PWH.

In this context, increasing attention has been directed toward modifiable risk factors in PWH, including dietary intake and overall diet quality. Diet is a key determinant of cardiometabolic health, and in recent years, the role of ultra-processed foods (UPF) has garnered growing interest. UPF are industrially manufactured products formulated from refined food components, additives, and processing aids, designed to enhance palatability, convenience, and shelf stability [3]. To characterize dietary intake beyond nutrient composition alone, several food and beverages classification systems have been proposed and developed, but the most extensively used system is the NOVA classification system [4], which categorizes foods into four groups according to their degree of industrial processing, ranging from unprocessed or minimally processed foods (group 1) to ultra-processed foods (group 4) [5].

Contemporary dietary patterns have increasingly shifted toward ready-to-eat and convenience foods, with UPF now accounting for a substantial proportion of total energy intake in many populations. High UPF consumption has been consistently associated with adverse health outcomes in the general population, including increased risk of cardiovascular events, metabolic dysfunction, and all-cause mortality [3,6,7]. Several biological mechanisms have been proposed to explain these associations, including disruption of the gut microbiome, impaired gut barrier integrity, altered satiety signaling, hormonal dysregulation, and exposure to food additives [8]. These pathways may promote chronic inflammation, oxidative stress, and endothelial dysfunction, leading to atherogenesis and CVD.

Despite growing evidence in HIV-uninfected populations, data examining the relationship between UPF intake and inflammation in PWH remains limited. Existing dietary studies in HIV have largely relied on nutrient-based analyses or composite diet quality indices, which may not fully capture the health effects of food processing. The NOVA framework offers a complementary approach by focusing on the degree of industrial processing, thereby capturing dietary exposures not reflected in traditional nutritional metrics.

Although few studies have directly evaluated UPF consumption in PWH, available evidence suggests that diet quality in this population is frequently suboptimal, with a substantial proportion classified as having “poor” or “suboptimal” dietary patterns [9]. Given the convergence of persistent immune activation, elevated baseline cardiovascular risk, and suboptimal diet quality, understanding the role of food processing in shaping inflammatory and cardiometabolic profiles in PWH is of particular importance.

Accordingly, the objective of this study was to evaluate the association between the degree of dietary food processing and markers of systemic inflammation, gut integrity, cardiovascular risk, and body composition in virologically suppressed PWH. We hypothesized that a higher proportion of energy intake derived from ultra-processed foods would be associated with an unfavorable inflammatory profile, greater cardiovascular burden, and adverse body composition. Additionally, we aimed to characterize overall diet quality in this population using the NOVA classification system.

## 2. Materials and Methods

### 2.1. Study Design and Population

This is a cross-sectional study at the University Hospitals Cleveland Medical Center (UHCMC), Cleveland, Ohio. We included adult individuals with a virologically suppressed HIV infection (Viral load < 400 copies/mL for 6 months or more) under a stable ART regimen. All participants completed a baseline clinical assessment, detailed history taking, specific metabolic, vascular, gut integrity, and inflammatory marker measurements.

### 2.2. Ethical Considerations

The Institutional Review Boards (IRB) of University Hospitals Cleveland Medical Center approved our study. An IRB-approved written informed consent was obtained from all participants prior to any study-related activity.

### 2.3. Study Measurements

#### 2.3.1. Baseline Characteristics of Participants

Well-trained healthcare professionals collected comprehensive data on participants using standardized questionnaires, including demographic characteristics, lifestyle factors (smoking, alcohol consumption, physical activity), and medical history.

#### 2.3.2. Dietary Measures

We assessed dietary intake using 24 h food and supplement recalls, conducted by trained personnel from a specialized nutrition research core under the supervision of a registered dietitian. Nutrition Data System for Research software (NDSR version 2018) was used to analyze nutritional data and collect nutritional variables including: total energy, fat, carbohydrate, protein, saturated fatty acids (SFA), monounsaturated fatty acids (MUFA), polyunsaturated fatty acids (PUFA), dietary fiber, soluble dietary fiber, insoluble dietary fiber, added sugars (by available carbohydrate), and added sugars (by total sugars). We also used the Healthy Eating Index (HEI) scores automatically calculated by the NDSR software based on each participant’s reported intake. To evaluate the level of food processing, we applied the NOVA classification system, which categorizes foods into one of the four groups according to the extent and purpose of industrial processing [10]. Following categorization, the percentage of total energy intake derived from each group was calculated. Dietary intake was summarized, and the percentage of total energy derived from NOVA 1 and NOVA 4 foods was calculated relative to total energy intake across all NOVA categories (NOVA 1–4). Based on the NOVA 4 median in our sample, we divided participants into two groups: unhealthy and healthier.

#### 2.3.3. Body Composition

Body composition was assessed by a whole-body dual-energy X-ray absorptiometry (DXA) along with a non-contrast helical computed tomography (CT) of the abdomen as previously detailed [11]. DXA scans were conducted using a standardized anteroposterior protocol on a single device (Lunar Prodigy Advance, GE Healthcare, Chicago, IL, USA) to quantify fat distribution (total body, limb, and trunk) and lean body mass (LBM). For abdominal fat assessment, CT imaging was performed with 3 mm slice increments spanning from the diaphragm to the symphysis pubis. A single axial image at the L4–L5 vertebral level was selected to estimate abdominal adipose tissue (AT) areas, including visceral (VAT), expressed in cm^2^. A single radiologist interpreted all imaging results to ensure consistency across measurements.

#### 2.3.4. Metabolic and Cardiovascular Biomarkers

All participants underwent blood pressure and anthropometric measurements, including hip and waist circumference, weight, and height. Serum metabolic measurements included levels of glucose, glycated hemoglobin A1C (HbA1C), triglycerides (TG), insulin levels to compute the homeostasis model assessment of insulin resistance (HOMA-IR), total cholesterol, very-low-density lipoprotein (VLDL), low-density lipoprotein (LDL), high-density lipoprotein (HDL), and non-HDL cholesterol. We used the EndoPAT^®^-2000 device (Itamar Medical, Caesarea, Israel) as an indirect and non-invasive tool to assess endothelial function, as we detailed in a previous study, generating a reactive hyperemic index (RHI, normal is >1.67) and an augmentation index corrected to a heart rate of 75 beats per minute (AI 75, lower values reflecting better elasticity) [12]. We calculated the 10-year atherosclerotic cardiovascular disease (ASCVD) score at baseline and 24 weeks using the ASCVD Risk Estimator Plus of the American College of Cardiology [13]. We used the Joint Interim Statement of 2009 to define metabolic syndrome (MetS) [14].

#### 2.3.5. Biomarkers

HIV-1 Ribonucleic Acid (HIV-1 RNA) and CD4 counts were obtained from the clinical charts, as these are part of routine HIV care. Additional plasma samples were promptly processed and stored at −80 °C within 2 h and shipped in batches without prior thawing to Dr. Funderburg’s laboratory at Ohio State University for the measurement of biomarkers of inflammation, monocyte activation, and gut integrity using enzyme-linked immunosorbent assays (ELISA). Monocyte activation and inflammatory and endothelial biomarkers included soluble CD14 and CD163 (sCD14 and sCD163), high-sensitivity C-reactive protein (hsCRP), interleukin-6 (IL-6), interferon-gamma inducible protein 10 (IP-10), soluble tumor necrosis factor receptors 1 and 2 (sTNF-RI and sTNF-RII), intercellular adhesion molecule (ICAM), and vascular cell adhesion molecule (VCAM) using ELISA kits from R&D Systems (Minneapolis, MN, USA). Levels of D-dimer and oxidized low-density lipoprotein (oxLDL) were measured using ELISA kits from Diagnostica Stago (Parsippany, NJ, USA) and Uppsala (Mercodia, Uppsala, Sweden), respectively. Gut biomarkers that were measured included zonulin (Immundiagnostik AG, Bensheim, Germany) and intestinal fatty-acid-binding protein (I-FABP, R&D Systems), and markers of bacterial and fungal translocation included lipopolysaccharide-binding protein (LBP, R&D Systems) and β-d-glucan (BDG, MBS756415, MyBioSource, San Diego, CA, USA), respectively.

### 2.4. Statistical Analysis

Continuous variables are presented as means ± standard deviation (SD), while categorical variables are presented as counts and percentages. Baseline characteristics and diet composition are presented according to percent NOVA 4 intake into 2 groups: the unhealthy group defined as NOVA 4 4 ≥ 45.56% vs. the healthier group defined as %NOVA 4 < 45.56%.

Univariate associations were examined using force-directed network plots, with separate networks constructed for body composition, inflammatory, gut, and cardiovascular biomarkers. In these networks, nodes represented individual variables, and edges indicated statistically significant correlations between them. With edge color reflecting direction (green = positive; red = negative), and edge thickness representing the strength of association.

To examine multivariate relationships and the impact of diet composition on body composition variables, gut biomarkers, inflammatory markers, and cardiovascular biomarkers, we employed generalized additive models (GAMs). The primary predictors were the percentage of dietary NOVA 1 and NOVA 4 foods, with age, absolute CD4, race, and sex included as covariates. This modeling framework allowed for the estimation of both linear and non-linear associations between predictors and continuous outcome variables.

GAMs were selected to flexibly accommodate potential non-linear relationships using penalized smooth functions fit using basis dimensions (*k*) between 3 and 5. Exploratory GAMs were initially fit with smooth terms for continuous predictors to assess evidence of nonlinearity. Functional form was evaluated based on estimated degrees of freedom (EDF), penalization behavior, and the stability of fitted smooth terms across model specifications. Predictors exhibiting minimal departure from linearity (e.g., EDF near 1 or weak curvature without meaningful improvement in model fit) were modeled as linear terms to promote parsimony and interpretability.

Linear associations are reported as β coefficients with 95% confidence intervals (CIs). Non-linear effects are summarized using EDF values and visualized with smooth spline functions and corresponding 95% CIs. Model diagnostics included inspection of residuals, assessment of smoothing adequacy, and evaluation of collinearity among predictors to ensure appropriate model fit.

Given the number of outcomes examined, correction for multiple testing was applied to the primary dietary exposure variables in the GAM analyses using the Benjamini–Hochberg false discovery rate (FDR) procedure. Statistical significance was defined as a two-sided FDR-adjusted q < 0.05 for GAMs.

## 3. Results

### 3.1. Participants’ Characteristics

A total of 222 participants met eligibility criteria and were categorized into 112 individuals in the unhealthy group and 110 in the healthier group based on median %NOVA 4 intake. Overall, the study population was 31.1% female, and 66.2% non-white race (Table 1). Asthma/Chronic obstructive pulmonary disease (COPD) was more common in the unhealthy group compared with the healthier group (37.5% vs. 22.7%, *p* = 0.017, alcohol consumption was significantly more frequent in the healthier group (79.1% vs. 60.7%, *p* = 0.008), and mean CD4 count was higher among individuals in the unhealthy group (818.1 vs. 703.8 cells/mm^3^, *p* = 0.033). The two groups were well balanced across all other demographic characteristics, past medical history, and HIV-related variables (Table 1; *p* > 0.05).

### 3.2. Diet Composition

The mean total energy intake of our sample was 4259 kcal and it was higher in the healthier group compared with the unhealthy group (4574 vs. 3949 kcal) (Table 2). The median proportion of total energy derived from ultra-processed foods (NOVA 4) in the unhealthy group was greater compared with the healthier group (57% (IQR 50–66) vs. 36% (28–43)), whereas the healthier group derived a higher proportion of energy from NOVA 1 foods (27% (19–37) vs. 18% (11–25)). The unhealthy group consumed greater amounts of total added sugars than the healthier group (201.84 ± 181.56 vs. 104.16 ± 89.04 g/day), while total dietary fiber intake was lower in the unhealthy group (24.47 ± 17.13 vs. 40.02 ± 71.31 g/day).

### 3.3. Associations Between UPF Intake and Body Composition

**Figure A1:**
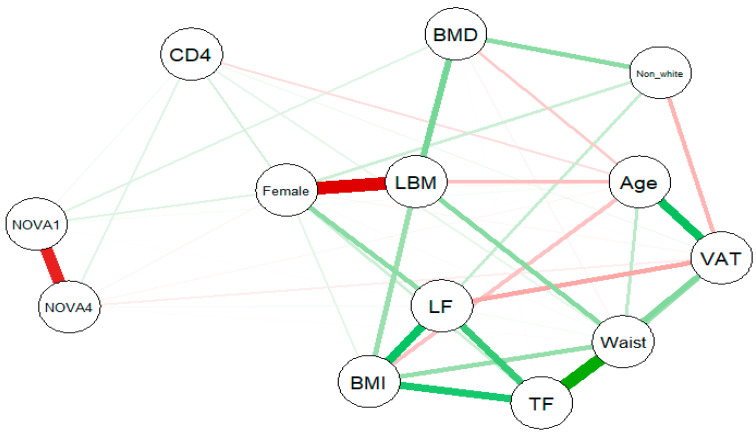
Force-directed network plot illustrating relationships among body composition variables connected by edges (green and red lines), representing the relationships between them. Edge color indicates the direction of the relationship (green, positive; red, negative), with the thickness and boldness of the color indicating the strength of the relationship. (LF = Limb fat, TF = Trunk Fat, LBM = Lean Body Mass, VAT = Estimated Visceral Adipose Tissue Area, Waist = Waist Circumference, BMD = Bone Mineral Density; BMI = Body Mass Index, NOVA 1 = Percent total energy intake of NOVA 1; NOVA 4 = Percent total energy intake of NOVA 4).

Body composition was strongly associated with age and sex, with weaker univariate associations observed for diet composition (Figure A1). These patterns remained consistent in multivariate models adjusting for age, sex, race, and CD4 count, in which percent energy intake from NOVA 4 foods was not associated with body mass index (BMI), fat mass, lean body mass, trunk fat, or estimated visceral adipose tissue area (Table 3). A significant non-linear association was observed between percent energy intake from NOVA 4 foods and waist circumference in unadjusted analyses (*p* = 0.04); however, this association did not remain statistically significant after false discovery rate (FDR) correction (Table 3; Figure A4). Non-linear associations were observed between age and several body composition outcomes, including waist circumference and bone mineral density (Table A1).

### 3.4. Associations Between UPF Intake and Inflammatory and Gut Biomarkers

**Figure 1:**
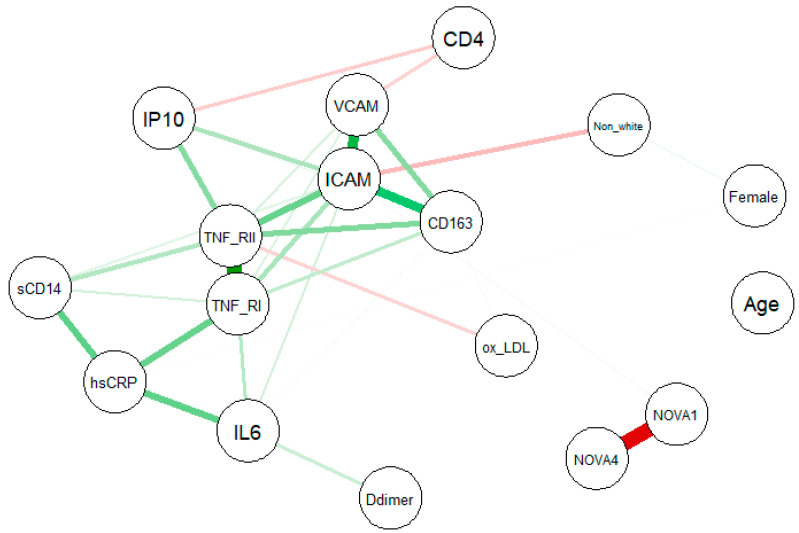
Force-directed network plotting displaying nodes (inflammation markers) connected by edges (green and red lines), representing the relationships between variables. Edge color indicates the direction of the relationship (green, positive; red, negative), with the thickness and boldness of the color indicating the strength of the relationship. (NOVA 1 = Percent total energy intake of NOVA 1; NOVA 4 = Percent total energy intake of NOVA 4).

**Figure A2:**
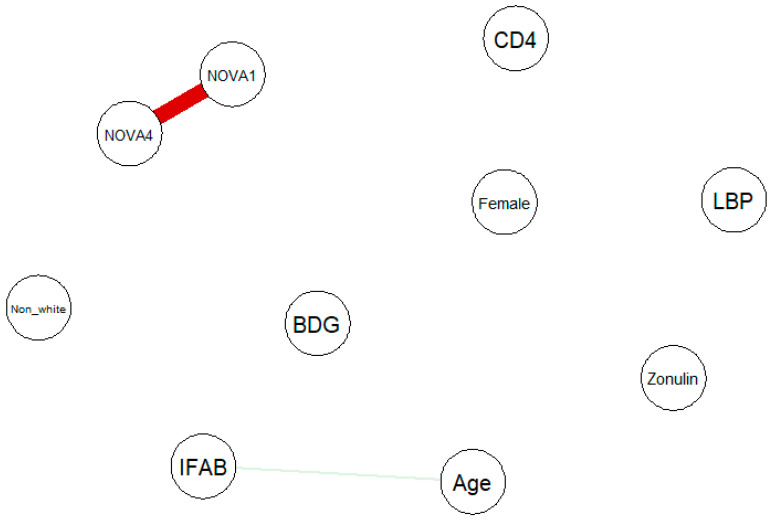
Force-directed network plotting displaying nodes (gut biomarkers) connected by edges (green and red lines), representing the relationships between them. Edge color indicates the direction of the relationship (green, positive; red, negative), with the thickness and boldness of the color indicating the strength of the relationship (NOVA 1 = Percent total energy intake of NOVA 1; NOVA 4 = Percent total energy intake of NOVA 4).

Univariate associations between study variables and inflammatory markers (Figure 1) and gut biomarkers (Figure A2) were generally weak. In multivariable models adjusting for age, sex, race, and CD4 count, percent energy intake from NOVA 4 foods was not significantly associated with markers of gut permeability or microbial translocation, including zonulin, IFABP, LBP, and BDG (Table 4). Likewise, percent energy intake from NOVA 4 foods was not independently associated with systemic inflammation or immune activation markers, including hsCRP, IL 6, IP 10, oxLDL, sCD14, sCD163, TNF RI, TNF RII, ICAM, VCAM, or D dimer (Table 4). A significant non-linear association was observed between percent energy intake from NOVA 1 foods and IP-10 (*p* = 0.002); however, this association was no longer statistically significant after FDR (Table 4; Figure A5). Instead, gut biomarkers and inflammatory markers were primarily associated with age, race, sex, and/or CD4 count (Table A2).

### 3.5. Associations Between UPF Intake and Cardiometabolic Outcomes

**Figure A3:**
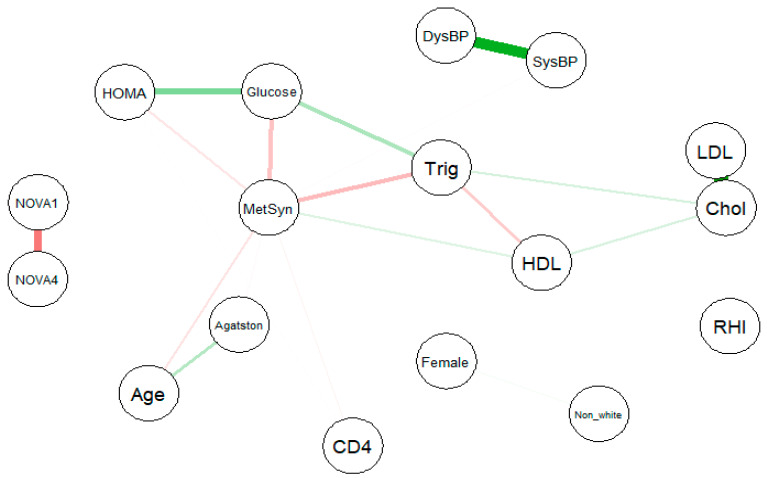
Force-directed network plotting displaying nodes (cardiovascular markers) connected by edges (green and red lines), representing the relationships between them. Edge color indicates the direction of the relationship (green, positive; red, negative), with the thickness and boldness of the color indicating the strength of the relationship (Chol = Total cholesterol; Trig = Total Triglycerides, MetSyn = Metabolic Syndrome (3 or more components); Agatston = Agatston Score; HOMA = HOMA-IR; SysBP = systolic blood pressure and DysBP = diastolic blood pressure.

Univariate associations between age and cardiometabolic markers were weak for Agatston score and metabolic syndrome, while all other markers showed no meaningful univariate associations with study variables (Figure A3). Similarly, multivariate models assessing cardiometabolic parameters, percent energy intake from NOVA 4 foods, were not associated with lipid measures, glucose, HOMA-IR, blood pressure, RHI, or 10-year ASCVD risk (Table 5). There was a significant negative association between cholesterol and %NOVA 4 in unadjusted models (*p* = 0.02); however, this association was no longer significant after FDR adjustment (Table 5). Instead, cardiometabolic markers were more strongly associated with CD4 and/or age (Table A3).

**Figure A4:**
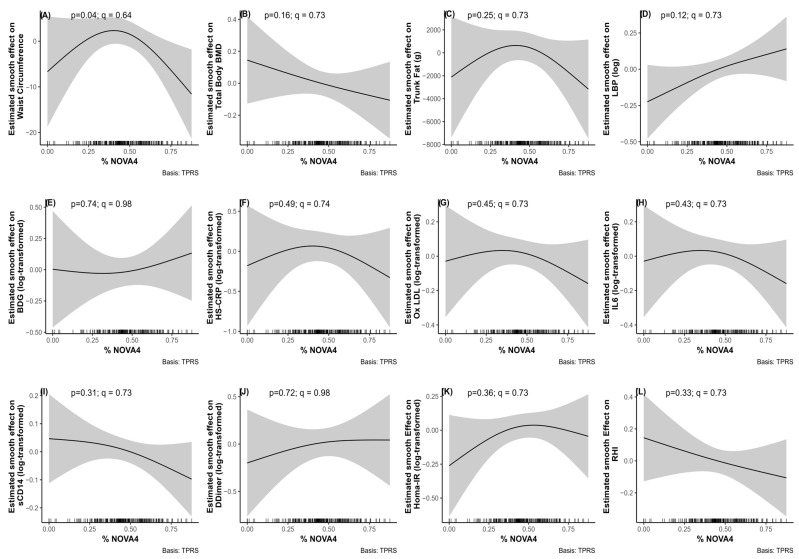
Estimated smooth (partial) effects of % NOVA 4 A (ultra-processed) foods on (**A**) waist circumference, (**B**) total body bone mineral density (BMD), (**C**) trunk fat (g), (**D**) LBP (log transformed), (**E**) BDG (log transformed), (**F**) HS-CRP (log-transformed), (**G**) OX_LDL (log-transformed), (**H**) IL6 (log-transformed), (**I**) sCD14 (log-transformed), (**J**) D-Dimer (log-transformed), (**K**) Home-IR (log-transformed), and (**L**) RHI from generalized additive models (GAMs). The association between % NOVA 4 intake and each outcome was modeled using a thin plate regression spline (k = 3), while age, absolute CD4 count, % NOVA 1 intake, race, and sex were included as covariates. Shaded bands represent 95% confidence intervals, and rug marks indicate the distribution of observed NOVA values. Statistical significance is shown using unadjusted *p*-values and FDR-adjusted *q*-values, as indicated.

**Figure A5:**
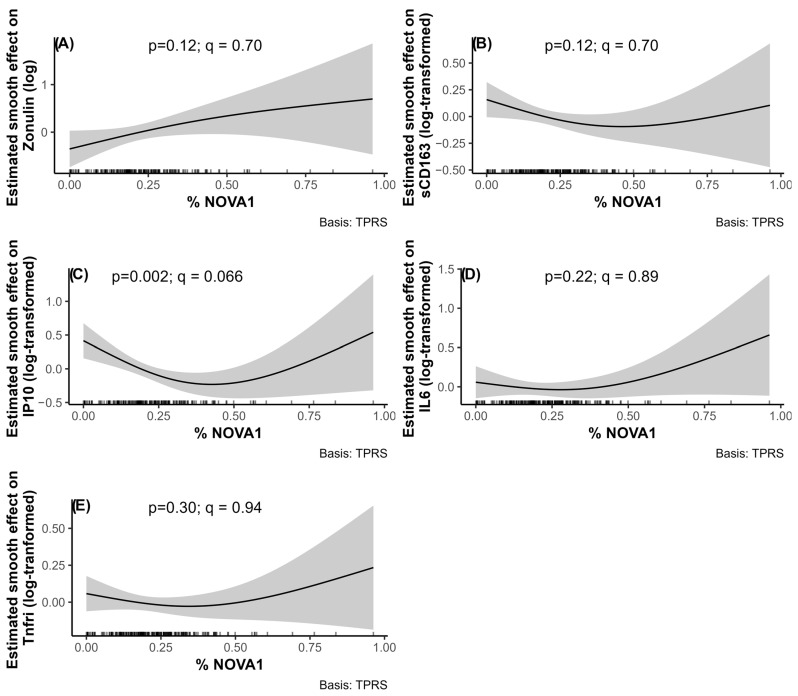
Estimated smooth (partial) effects of % NOVA 1 (not processed) foods on (**A**) Zonulin (log-transformed), (**B**) sCD163 (log-transformed), (**C**) IP-10 (log-transform) (**D**) IL-6 (log-transformed) and (**E**) TNF-RI (log-transformed) from generalized additive models (GAMs). The association between % NOVA 1 intake and each outcome was modeled using a thin plate regression spline (k = 3), while age, absolute CD4 count, % NOVA 4 intake, race, and sex were included as covariates. Shaded bands represent 95% confidence intervals, and rug marks indicate the distribution of observed NOVA values. Statistical significance is shown using unadjusted *p*-values and FDR-adjusted *q*-values, as indicated.

Nonsignificant non-linear associations between other variables and percent energy intake from NOVA 4 and NOVA 1 foods are represented in Figure A4 and Figure A5, respectively.

## 4. Discussion

In this cross-sectional study of virologically suppressed PWH on stable ART, we found that a higher proportion of dietary energy derived from UPF was not independently associated with systemic inflammation, immune activation, gut permeability, cardiometabolic risk, or adverse body composition after adjustment for key demographic and HIV-related factors. Additionally, we found substantial consumption of UPF in this cohort. These findings contrast with observations from the general population and suggest that the pathways linking UPF food intake to adverse cardiometabolic health may operate differently in the context of treated HIV infection. A recently published systematic review and meta-analysis have shown that the association between ultra-processed food consumption and all causes and cardiovascular mortality was explained, amongst other factors, by the increased inflammatory markers [15]. Hence, the results of our study were unexpected, given that the previous evidence suggests that among non-HIV patients, UPF intake is associated with higher inflammation. One possible explanation for these findings is that inflammation in PWH might be influenced by persistent immune activation, microbial translocation, and ART-related factors, which may outweigh potential dietary influences, even in the setting of virologic suppression [16,17]. However, this interpretation remains hypothesis-generating and requires confirmation in longitudinal and mechanistic studies.

A similar study in Spain observed that individuals with HIV on stable ART with an undetectable HIV viral load, consuming a Western-like dietary pattern, had significantly higher levels of inflammatory biomarkers, including D-dimer (*p* = 0.050) and soluble TNF-alpha receptor 2 (sTNFR2) (*p* = 0.049), compared to those following a Mediterranean-like dietary pattern [18]. In contrast, the present study found no significant association between dietary patterns and inflammatory markers in our cohort of people living with HIV. This discrepancy may be attributable to differences in statistical power, as Manzano et al. studied 27 participants, whereas our larger sample size of 222 participants provided greater power to detect associations [18]. Alternatively, the null findings in our study could reflect differences in population characteristics, dietary assessment methods, or the specific inflammatory markers examined.

Weiss et al. reported that, among 103 individuals with HIV, higher diet quality scores were associated only with lower log-transformed concentrations of sCD14; diet quality was not correlated with other markers of immune activation, including hsCRP, sCD163, IL-6, and MCP-1 [19]. The study further demonstrated that diet quality was significantly lower in women with HIV compared with men (HEI score 49.2 vs. 55.7, *p* = 0.005), suggesting sex-specific differences in dietary patterns and their potential inflammatory effects [19]. Although we did not conduct sex-stratified analyses, these findings are consistent with our results, which showed that percent energy intake from NOVA 4 foods was not independently associated with elevated inflammatory markers.

Furthermore, a clinical trial assessing rosuvastatin for cardiovascular disease risk examined dietary factors, gut integrity, and inflammation in 147 adults with HIV, and found that alcohol consumption was associated with poorer gut integrity markers [20]. Specifically, liquor consumption in the previous week was positively correlated with higher LBP. Additionally, alcohol consumption was associated with increased inflammation, while other dietary components (fiber, carbohydrates, fat) showed no significant associations [20]. The lack of associations with most dietary factors is consistent with our findings; however, alcohol-related gut biomarkers and inflammatory outcomes were not evaluated in the present study.

Although this study has many strengths, particularly, it has a relatively large sample compared to other studies addressing a similar topic. Also, our study included a comprehensive assessment of inflammation and gut markers, dietary intake, and body composition measures. There are, however, some limitations that should be acknowledged. First, while NOVA provides a framework for assessing the role of industrial food processing in health outcomes, it is not without limitations. Food classification is inherently subjective, often requiring a nuanced interpretation of ingredient lists and manufacturing processes. Moreover, the system does not account for variability in nutrient density, micronutrient fortification, or culinary context across foods within the same processing group [21]. These limitations introduce challenges to reproducibility and generalizability across studies. Nevertheless, NOVA remains a widely utilized tool in nutritional epidemiology for capturing dimensions of diet not reflected in conventional nutrient-based indices [22]. Second, given the cross-sectional nature of this study, causality cannot be proven as it represents a snapshot in time rather than a cause-and-effect relation. Additionally, although major confounders such as age, sex, race, and CD4 count were adjusted for, residual confounding from lifestyle factors, medications, or unmeasured variables cannot be excluded. Our study population was recruited from a single U.S. center, potentially limiting generalizability to other regions or populations with different genetic, nutritional, or environmental backgrounds. Lastly, the self-reported UPF intake based on a single 24 h diet recall is subject to social desirability and underreporting bias.

## 5. Conclusions

In a cohort of virologically suppressed PWH, a higher proportion of energy intake from UPF was not associated with enhanced systemic inflammation, immune activation, gut permeability, cardiometabolic risk, or adverse body composition. Longitudinal and larger studies incorporating repeated dietary assessments and interventional trials are warranted to clarify the role of food processing in long-term cardiometabolic risk in this population.

## Figures and Tables

**Table 1:** Baseline characteristics in HIV patients stratified by NOVA 4 dietary intake below and above the median of 45.56%.

Characteristic	Overall N = 222 ^1^	Healthier N = 110 ^1^	Unhealthy N = 112 ^1^	*p*-Value ^2^
**Age**	45.4 ± 14.2	46.9 ± 14.2	44.0 ± 14.0	0.13
**Sex**				0.269
Female	69 (31.1%)	38 (34.5%)	31 (27.7%)	
Male	153 (68.9%)	72 (65.5%)	81 (72.3%)	
**Race**				0.339
African American	137 (61.7%)	61 (55.5%)	76 (67.9%)	
Asian	1 (0.5%)	1 (0.9%)	0 (0.0%)	
Bi-Racial	4 (1.8%)	3 (2.7%)	1 (0.9%)	
Caucasian	75 (33.8%)	41 (37.3%)	34 (30.4%)	
Native American	1 (0.5%)	1 (0.9%)	0 (0.0%)	
Other	1 (0.5%)	1 (0.9%)	0 (0.0%)	
**Ethnicity**				0.785
Hispanic or Latino	21 (9.5%)	11 (10.0%)	10 (8.9%)	
Non-Hispanic or Non-Latino	201 (90.5%)	99 (90.0%)	102 (91.1%)	
**Diagnoses: Past Medical + Current**				
Hypertension	76 (34.2%)	40 (36.4%)	36 (32.1%)	0.508
Hyperlipidemia	36 (16.2%)	17 (15.5%)	19 (17.0%)	0.76
High cholesterol	34 (15.3%)	18 (16.4%)	16 (14.3%)	0.667
Diabetes	11 (5.0%)	4 (3.6%)	7 (6.3%)	0.37
Asthma/COPD	67 (30.2%)	25 (22.7%)	42 (37.5%)	**0.017**
CD4 < 200	37 (16.7%)	22 (20.0%)	15 (13.4%)	0.187
Malignancy	12 (5.4%)	8 (7.3%)	4 (3.6%)	0.223
Substance abuse	49 (22.1%)	23 (20.9%)	26 (23.2%)	0.679
**Smoking Status**				0.455
Current	105 (47.3%)	50 (45.5%)	55 (49.1%)	
Never	74 (33.3%)	35 (31.8%)	39 (34.8%)	
Past	43 (19.4%)	25 (22.7%)	18 (16.1%)	
**Alcohol Status**				**0.008**
Current	155 (69.8%)	87 (79.1%)	68 (60.7%)	
Never	19 (8.6%)	5 (4.5%)	14 (12.5%)	
Past	48 (21.6%)	18 (16.4%)	30 (26.8%)	
**HIV Condition**				
CD4 Absolute (cells/mm^3^)	762.3 ± 398.7	703.8 ± 375.1	818.1 ± 414.1	**0.033**
HIV duration (months)	165.9 ± 123.7	172.2 ± 128.7	159.6 ± 118.8	0.447
Viral load (copies/mL)	20 (20, 20)	20 (20, 20)	20 (20, 20)	0.417
**BMI (Kg/m^2^)**	30.61 ± 7.91	30.39 ± 7.72	30.82 ± 8.12	0.691

^1^ Mean ± SD; n (%); median (Q1, Q3). ^2^ Welch Two Sample *t*-test; Pearson’s Chi-squared test; Fisher’s exact test; Wilcoxon rank sum test. COPD: Chronic Obstructive Pulmonary Disorder; BMI: Body Mass Index; HIV: Human Immunodeficiency Virus. Statistically significant *p*-values (*p* < 0.05) are shown in bold.

**Table 2:** Overall diet composition in HIV patients stratified by NOVA 4 dietary intake below and above the median of 45.56%.

Characteristic	Overall N = 222 ^1^	Healthier N = 110 ^1^	Unhealthy N = 112 ^1^
Total Energy (kcal)	4258.95 ± 4274.96	4574.03 ± 5573.90	3949.49 ± 2383.77
Total Fats (g)	187.59 ± 289.27	213.91 ± 395.35	161.75 ± 108.40
Total Carbohydrates (g)	470.69 ± 383.02	447.93 ± 444.58	493.04 ± 311.41
Total Proteins (g)	161.00 ± 162.39	184.05 ± 211.96	138.36 ± 85.67
Total Saturated Fats (g)	59.63 ± 60.00	65.35 ± 76.03	54.01 ± 37.78
Total Monounsaturated Fats (g)	64.17 ± 86.64	73.63 ± 116.93	54.88 ± 36.65
Total polyunsaturated Fats (g)	48.94 ± 146.07	58.02 ± 205.07	40.03 ± 32.01
Total Dietary Fibers (g)	32.18 ± 52.12	40.02 ± 71.31	24.47 ± 17.13
Total Soluble Dietary Fibers (g)	10.27 ± 10.47	11.70 ± 12.95	8.88 ± 7.06
Total Insoluble Dietary Fibers (g)	20.72 ± 40.06	26.01 ± 55.32	15.52 ± 11.60
Total Added Sugars (carbs) (g)	169.74 ± 163.71	113.52 ± 98.12	224.96 ± 194.16
Total Added Sugars (Total Sugars) (g)	153.44 ± 151.20	104.16 ± 89.04	201.84 ± 181.56
NOVA 1 (%)	21% (14, 30)	27% (19, 37)	18% (11, 25)
NOVA 4 (%)	46% (36, 58)	36% (28, 43)	57% (50, 66)

^1^ Mean ± SD; n (%); median (Q1, Q3).

**Table 3:** Separate generalized additive models (GAMs) examining associations between body composition and dietary predictors (% NOVA 1 and % NOVA 4).

Body Composition	Estimate: Beta/EDF ^1^	95% CI	*p*-Value	q-Value ^2^	Adjusted R^2 3^
**Weight**					
% NOVA 1	5.4	−20, 31	0.68	0.94	
% NOVA 4	−8.3	−30, 14	0.46	0.73	0.095
**BMI**					
% NOVA 1	1.0	−7.2, 9.2	0.81	0.94	
% NOVA 4	−1.6	−8.7, 5.5	0.66	0.97	0.156
**Waist circumference**					
% NOVA 1	6.9	−13, 26	0.48	0.94	
% NOVA 4 (non-linear)	1.85	-	**0.04**	0.64	0.183
**Total Body Bone Mineral Density**					
% NOVA 1	0.10	−0.02, 0.22	0.12	0.69	
% NOVA 4 (non-linear)	1.78	-	0.16	0.73	0.172
**Lean body mass**					
% NOVA 1	2817	−8175, 13,808	0.61	0.94	
% NOVA 4	−5278	−14,785, 4229	0.27	0.73	0.28
**Total Limb fat**					
% NOVA 1	431	−6466, 7328	0.90	0.94	
% NOVA 4	144	−5822, 6109	0.96	0.99	0.257
**Trunk fat**					
% NOVA 1	1429	−7496, 10,355	0.75	0.94	
% NOVA 4 (non-linear)	1.72	-	0.25	0.73	0.18
**Estimated VAT area**					
% NOVA 1	−8.6	−67, 50	0.77	0.94	
% NOVA 4	−35.05	−86, 16	0.25	0.73	0.255

^1^ For linear terms, estimates represent regression coefficients (β). For non-linear terms, estimates represent estimated degrees of freedom (EDF). ^2^ q-values represent *p*-values adjusted for multiple comparisons using the Benjamini–Hochberg false discovery rate (FDR) procedure, applied separately for each NOVA exposure across outcomes. ^3^ Adjusted R^2^ corresponds to the full multivariate generalized additive model, which included age, absolute CD4 count, sex, and race as covariates (see Table A1, Table A2 and Table A3). BMI: Body Mass Index; VAT: Visceral Adipose Tissue. Statistically significant *p*-values (*p* < 0.05) are shown in bold.

**Table 4:** Separate generalized additive models (GAMs) examining associations between gut and inflammation markers and dietary predictors (% NOVA 1 and % NOVA 4).

Gut and Inflammation Marker	Estimate: Beta/EDF ^1^	95% CI	*p*-Value	q-Value ^2^	Adjusted R^2 3^
**Zonulin (log)**					
% NOVA 1 (non-linear)	1.32	-	0.12	0.70	
% NOVA 4	−0.07	−1.3, 1.1	0.91	0.99	0.099
**IFABP (log)**					
% NOVA 1	−0.06	−0.74, 0.62	0.86	0.94	
% NOVA 4	0.09	−0.50, 0.68	0.76	0.98	0.091
**LBP (log)**					
% NOVA 1	0.40	−0.12, 0.92	0.13	0.70	
% NOVA 4	1.31	-	0.12	0.73	0.12
**BDG (log)**					
% NOVA 1	0.30	−0.59, 1.2	0.51	0.94	
% NOVA 4 (non-linear)	1.33	-	0.74	0.98	−0.006
**hsCRP (log)**					
% NOVA 1	0.12	−1.2, 1.5	0.86	0.94	
% NOVA 4 (non-linear)	1.57	-	0.49	0.74	0.05
**OxLDL (log)**					
% NOVA 1	−0.12	−0.68, 0.45	0.68	0.94	
% NOVA 4 (non-linear)	-	-	0.45	0.73	0.089
**TNF-RI (log)**					
% NOVA 1 (non-linear)	1.72	-	0.30	0.94	
% NOVA 4	0.30	−0.03, 0.64	0.074	0.73	0.085
**TNF-RII (log)**					
% NOVA 1	−0.12	−0.54, 0.30	0.58	0.94	
% NOVA 4	0.14	−0.23, 0.51	0.45	0.73	0.086
**IL-6 (log)**					
% NOVA 1 (non-linear)	1.82	-	0.22	0.89	
% NOVA 4 (non-linear)	1.58	-	0.43	0.73	0.036
**IP-10 (log)**					
% NOVA 1 (non-linear)	1.91	-	**0.002**	0.066	
% NOVA 4	0.08	−0.63, 0.79	0.83	0.99	0.177
**ICAM-1 (log)**					
% NOVA 1	−0.68	−1.3, −0.07	**0.028**	0.45	
% NOVA 4	0.45	−0.09, 1.0	0.10	0.73	0.20
**VCAM-1 (log)**					
% NOVA 1	0.02	−0.32, 0.36	0.93	0.94	
% NOVA 4	0.13	−0.16, 0.42	0.39	0.73	0.219
**sCD14 (log)**					
% NOVA 1	−0.20	−0.51, 0.10	0.19	0.89	
% NOVA 4 (non-linear)	1.37	-	0.31	0.73	0.107
**sCD163 (log)**					
% NOVA 1 (non-linear)	−1.73	-	0.12	0.70	
% NOVA 4	0.03	−0.43, 0.49	0.90	0.99	0.05
**D-dimer (log)**					
% NOVA 1	0.20	−0.97, 1.4	0.74	0.94	
% NOVA 4 (non-linear)	1.24	-	0.72	0.98	0.041

^1^ For linear terms, estimates represent regression coefficients (β). For non-linear terms, estimates represent estimated degrees of freedom (EDF). ^2^ q-values represent *p*-values adjusted for multiple comparisons using the Benjamini–Hochberg false discovery rate (FDR) procedure, applied separately for each NOVA exposure across outcomes. ^3^ Adjusted R^2^ corresponds to the full multivariate generalized additive model, which included age, absolute CD4 count, sex, and race as covariates (see Table A1, Table A2 and Table A3). IL-6 = Interleukin-6; VCAM-1 = Vascular Cell Adhesion Molecule-1; ICAM-1 = Intercellular Adhesion Molecule-1; TNF-RI = Tumor Necrosis Factor Receptor-1; TNF-RII = Soluble Tumor Necrosis Factor Receptor-2; hsCRP = High-sensitivity C-reactive protein; IP-10: Interferon-gamma-induced protein 10; OxLDL = Oxidized Low-Density Lipoprotein; sCD14 = Soluble CD14; sCD163 = Soluble CD163; IFABP = Intestinal fatty-acid binding protein; LBP = Lipopolysaccharide binding protein; BDG: Beta-D-glucan. Statistically significant *p*-values (*p* < 0.05) are shown in bold.

**Table 5:** Separate generalized additive models (GAMs) examining associations between cardiometabolic biomarkers and dietary predictors (% NOVA 1 and % NOVA 4).

Cardiometabolic Biomarker	Estimate: Beta/EDF ^1^	95% CI	*p*-Value	q-Value ^2^	Adjusted R^2 3^
**Triglycerides (log)**					
% NOVA 1	−0.06	−0.66, 0.54	0.84	0.94	
% NOVA 4	−0.06	−0.58, 0.46	0.82	0.99	0.137
**Cholesterol (log)**					
% NOVA 1	−0.05	−0.30, 0.19	0.70	0.94	
% NOVA 4	−0.24	−0.45, −0.04	**0.02**	0.64	0.67
**non-HDL**					
% NOVA 1	−1.6	−42, 39	0.94	0.94	
% NOVA 4	−21	−56, 14	0.23	0.73	0.069
**LDL**					
% NOVA 1	2.2	−33, 37	0.91	0.94	
% NOVA 4	−15	−46, 15	0.33	0.73	0.031
**Glucose (log)**					
% NOVA 1	0.02	−0.17, 0.21	0.84	0.94	
% NOVA 4	0.01	−0.17, 0.16	0.99	0.99	0.021
**HOMA-IR (log)**					
% NOVA 1	0.26	−0.40, 0.91	0.44	0.94	
% NOVA 4 (non-linear)	1.62	-	0.36	0.73	0.06
**RHI**					
% NOVA 1	−0.18	−0.80, 0.45	0.78	0.94	
% NOVA 4 (non-linear)	1.10	-	0.33	0.73	0.041
**Systolic BP**					
% NOVA 1	4.7	−12, 22	0.58	0.94	
% NOVA 4	7.5	−7.0, 22	0.31	0.73	0.04
**Diastolic BP**					
% NOVA 1	−0.81	−11, 9.6	0.87	0.94	
% NOVA 4	−0.36	−9.4, 8.7	0.94	0.99	0.052

^1^ For linear terms, estimates represent regression coefficients (β). For non-linear terms, estimates represent estimated degrees of freedom (EDF). ^2^ q-values represent *p*-values adjusted for multiple comparisons using the Benjamini–Hochberg false discovery rate (FDR) procedure, applied separately for each NOVA exposure across outcomes. ^3^ Adjusted R^2^ corresponds to the full multivariate generalized additive model, which included age, absolute CD4 count, sex, and race as covariates (see Table A1, Table A2 and Table A3). BP: Blood Pressure; HOMA-IR: Homeostatic Model Assessment of Insulin Resistance; LDL = Low-Density Lipoprotein; HDL = High-Density Lipoprotein; RHI: Reactive Hyperemic Index. Statistically significant *p*-values (*p* < 0.05) are shown in bold.

## Data Availability

The original contributions presented in this study are included in the article. Further inquiries can be directed to the corresponding author.

## Abbreviations

The following abbreviations are used in this manuscript:

AI	Augmentation Index
ART	Antiretroviral therapy
ASCVD	Atherosclerotic cardiovascular disease
AT	Adipose tissue
CI	Confidence Interval
CT	Computed tomography
CVD	Cardiovascular disease
DXA	Dual-energy X-ray absorptiometry
EDF	Estimated degrees of freedom
ELISA	Enzyme-linked immunosorbent assays
GAM	Generalized additive models
HbA1c	Hemoglobin A1C
HDL	High-density lipoprotein
HEI	Healthy Eating Index
HOMA-IR	Homeostasis Model Assessment of Insulin Resistance
HIV	Human Immunodeficiency Virus
LBM	Lean body mass
LDL	Low-density lipoprotein
MUFA	Monounsaturated Fatty Acids
NDSR	Nutrition Data System for Research
PUFA	Polyunsaturated Fatty Acids
PWH	People with HIV
RHI	Reactive Hyperemic Index
SFA	Saturated Fatty Acids
TG	Triglycerides
UPF	Ulta-processed food
UHCMC	University Hospitals Cleveland Medical Center
VAT	Visceral adipose tissue
VLDL	Very-low-density lipoprotein

## Appendix A

**Table A1:** Separate generalized additive models (GAMs) examining associations between body composition variables and the covariates of absolute CD4 count (per 1000 units), race, sex, and age as covariates. Models also controlled %NOVA 1 and %NOVA 4 in diet.

Body Composition	Estimate: Beta/EDF ^1^	95% CI	*p*-Value
**Weight**			
CD4 Absolute (per 1000)	7.8	−0.10, 16	0.053
Race			
African American	—	—	
Asian	−26	−73, 20	0.3
Bi-Racial	8.4	−15, 31	0.5
Caucasian	−8	−15, −1.2	**0.022**
Hispanic	−31	−58, −4.8	**0.021**
Native American	24	−21, 70	0.3
Other	−27	−72, 19	0.3
Sex			
Female	—	—	
Male	2.5	−4.6, 9.6	0.5
Age (non-linear)	1.93	—	**0.002**
**BMI**			
CD4 Absolute (per 1000)	3.1	0.53, 5.6	**0.018**
Race			
African American	—	—	
Asian	−8.3	−23, 6.6	0.3
Bi-Racial	1.9	−5.5, 9.4	0.6
Caucasian	−1.9	−4.1, 0.27	0.084
Hispanic	−11	−20, −2.5	**0.011**
Native American	7.1	−7.5, 22	0.3
Other	−8.6	−23, 6.2	0.3
Sex			
Female	—	—	
Male	−4.2	−6.5, −1.9	**<0.001**
Age (non-linear)	1.98	—	**0.034**
**Waist circumference**			
CD4 Absolute (per 1000)	8	2.0, 14	**0.009**
Race			
African American	—	—	
Asian	−16	−52, 20	0.4
Bi-Racial	6.8	−11, 24	0.4
Caucasian	−3.2	−8.4, 2.1	0.2
Hispanic	−21	−41, −0.56	**0.044**
Native American	18	−16, 53	0.3
Other	−18	−53, 17	0.3
Sex			
Female	—	—	
Male	−7.4	−13, −2.0	**0.008**
Age (non-linear)	1.88	—	**0.001**
**Total Body Bone Mineral Density**			
CD4 Absolute (per 1000)	0.01	−0.03, 0.04	0.8
Race			
African American	—	—	
Asian	−0.13	−0.37, 0.09	0.2
Bi-Racial	0.01	−0.10, 0.11	>0.9
Caucasian	−0.07	−0.10, −0.04	**<0.001**
Hispanic	−0.08	−0.20, 0.04	0.2
Native American	−0.10	−0.32, 0.11	0.3
Other	−0.10	−0.31, 0.11	0.4
Sex			
Female	—	—	
Male	0.05	0.02, 0.09	**0.002**
Age (non-linear)	1.83	—	**0.001**
**Lean body mass**			
CD4 Absolute (per 1000)	3443	38, 6849	**0.048**
Race			
African American	—	—	
Asian	−10,695	−30,704, 9313	0.3
Bi-Racial	2617	−7291, 12,526	0.6
Caucasian	−3245	−6193, −298	**0.031**
Hispanic	−12,824	−24,221, −1428	**0.028**
Native American	9026	−10,489, 28,541	0.4
Other	−6996	−26,756, 12,764	0.5
Sex			
Female	—	—	
Male	12,284	9202, 15,366	**<0.001**
Age (non-linear)	1.95	—	**<0.001**
**Total Limb fat**			
CD4 Absolute (per 1000)	1127	−1010, 3264	0.3
Race			
African American	—	—	
Asian	−5997	−18,550, 6556	0.3
Bi-Racial	2315	−3903, 8532	05
Caucasian	−3011	−4859, −1162	**0.002**
Hispanic	−8261	−15,412, −1110	**0.024**
Native American	4106	−8139, 16,350	0.5
Other	−8869	−21,264, 3525	0.2
Sex			
Female	—	—	
Male	−5802	−7734, −3870	**<0.001**
Age (non-linear)	1.90	—	**0.016**
**Trunk fat**			
CD4 Absolute (per 1000)	2863	94, 5632	**0.043**
Race			
African American	—	—	
Asian	−4711	−21,102, 11,681	0.6
Bi-Racial	4042	−3977, 12,061	0.3
Caucasian	−1464	−3844, 916	0.2
Hispanic	−9427	−18,631, −224	**0.045**
Native American	7678	−8107, 23,463	0.3
Other	−9820	−25,760, 6120	0.2
Sex			
Female	—	—	
Male	−5441	−7925, −2958	**<0.001**
Age (non-linear)	1.82	—	**0.018**
**Estimated VAT area**			
CD4 Absolute (per 1000)	27	8.4, 45	**0.004**
Race			
African American	—	—	
Asian	−16	−122, 90	0.8
Bi-Racial	41	−11, 94	0.12
Caucasian	16	0.06, 31	**0.049**
Hispanic	−41	−102, 19	0.2
Native American	68	−37, 171	0.2
Other	−37	−142, 68	0.5
Sex			
Female	—	—	
Male	−1.7	−18, 15	0.8
Age (non-linear)	1.66	—	**<0.001**

^1^ For linear terms, estimates represent regression coefficients (β). For non-linear terms, estimates represent estimated degrees of freedom (EDF). BMI: Body Mass Index; VAT: Visceral Adipose Tissue. Statistically significant *p*-values (*p* < 0.05) are shown in bold.

**Table A2:** Separate generalized additive models (GAMs) examining associations between gut and inflammation markers and the covariates of absolute CD4 count (per 1000 units), race, sex, and age as covariates. Models also controlled for %NOVA 1 and %NOVA 4 in diet.

Gut and Inflammation Markers	Estimate: Beta/EDF ^1^	95% CI	*p*-Value
**Zonulin (log)**			
CD4 Absolute (per 1000)	0.3	−0.10, 0.70	0.14
Race			
African American	—	—	
Asian	−0.67	−2.9, 1.5	0.6
Bi-Racial	−0.55	−1.7, 0.54	0.3
Caucasian	−0.21	−0.57, 0.14	0.2
Hispanic	−0.3	−1.9, 1.3	0.7
Native American	−0.46	−2.6, 1.7	0.7
Other	−1.4	−3.6, 0.75	0.2
Sex			
Female	—	—	
Male	−0.3	−0.66, 0.06	0.1
Age (non-linear)	1.81	—	**0.016**
**IFABP (log)**			
CD4 Absolute (per 1000)	0.12	−0.09, 0.34	0.2
Race			
African American	—	—	
Asian	0.71	−0.53, 2.0	0.3
Bi-Racial	−0.48	−1.1, 0.14	0.13
Caucasian	−0.14	−0.33, 0.04	0.12
Hispanic	0.08	−0.63, 0.79	0.8
Native American	−0.69	−1.9, 0.52	0.3
Other	−0.31	−1.5, 0.91	0.6
Sex			
Female	—	—	
Male	0.16	−0.03, 0.35	0.1
Age (per 5 years)	0.07	0.04, 0.10	**<0.001**
**LBP (log)**			
CD4 Absolute (per 1000)	0.19	0.03, 0.35	**0.021**
Race			
African American	—	—	
Asian	−0.48	−1.4, 0.46	0.3
Bi-Racial	−0.34	−0.81, 0.13	0.2
Caucasian	−0.05	−0.19, 0.08	0.4
Hispanic	−0.3	−0.84, 0.24	0.3
Native American	0.93	0.01, 1.8	**0.048**
Other	−1.6	−2.6, −0.71	**<0.001**
Sex			
Female	—	—	
Male	−0.22	−0.36, −0.07	**0.003**
Age (non-linear)	0.01	−0.03, 0.02	0.8
**BDG (log)**			
CD4 Absolute (non-linear)	1.50	—	0.6
Race			
African American	—	—	
Asian	−0.82	−2.3, 0.66	0.3
Bi-Racial	−0.29	−1.0, 0.44	0.4
Caucasian	0.03	−0.20, 0.27	0.8
Hispanic	0.26	−0.79, 1.3	0.6
Native American	−0.49	−1.9, 0.95	0.5
Other	−0.87	−2.3, 0.57	0.2
Sex			
Female	—	—	
Male	−0.24	−0.48, 0.00	0.051
Age (per 5 years)	0.01	−0.03, 0.05	0.7
**hsCRP (log)**			
CD4 Absolute (per 1000)	0.01	−0.41, 0.42	>0.9
Race			
African American	—	—	
Asian	−2.4	−4.8, 0.08	0.058
Bi-Racial	−0.02	−1.2, 1.2	>0.9
Caucasian	0.08	−0.27, 0.44	0.7
Hispanic	−0.78	−2.2, 0.61	0.3
Native American	0.28	−2.1, 2.7	0.8
Other	−2.6	−5.0, −0.21	**0.033**
Sex			
Female	—	—	
Male	−0.61	−0.98, −0.24	**0.001**
Age	−0.01	−0.02, 0.00	0.2
**OxLDL (log)**			
CD4 Absolute (non-linear)	1.74	—	0.15
Race			
African American	—	—	
Asian	−0.03	−0.97, 0.90	>0.9
Bi-Racial	0.16	−0.30, 0.62	0.5
Caucasian	0.1	−0.05, 0.24	0.2
Hispanic	−0.84	−1.5, −0.18	**0.013**
Native American	0.84	−0.07, 1.8	0.069
Other	0.02	−0.88, 0.93	>0.9
Sex			
Female	—	—	
Male	−0.08	−0.23, 0.07	0.3
Age	0.01	0.00, 0.01	**0.005**
**TNF-RI (log)**			
CD4 Absolute (per 1000)	−0.02	−0.14, 0.10	0.8
Race			
African American	—	—	
Asian	0.28	−0.42, 0.99	0.4
Bi-Racial	0.13	−0.21, 0.48	0.4
Caucasian	0.22	0.12, 0.32	**<0.001**
Hispanic	0.29	−0.12, 0.69	0.2
Native American	0.02	−0.67, 0.71	>0.9
Other	−0.15	−0.84, 0.55	0.7
Sex			
Female	—	—	
Male	−0.11	−0.22, 0.00	0.05
Age (non-linear)	1.79	—	0.05
**TNF-RII (log)**			
CD4 Absolute (per 1000)	−0.21	−0.34, −0.08	**0.002**
Race			
African American	—	—	
Asian	0.17	−0.60, 0.94	0.7
Bi-Racial	−0.22	−0.60, 0.16	0.3
Caucasian	0.15	0.04, 0.27	**0.007**
Hispanic	0.26	−0.18, 0.70	0.2
Native American	−0.9	−1.6, −0.14	**0.02**
Other	−0.04	−0.80, 0.71	>0.9
Sex			
Female	—	—	
Male	−0.15	−0.26, −0.03	**0.014**
Age (per 5 years)	0.01	−0.01, 0.02	0.6
**IL-6 (log)**			
CD4 Absolute (per 1000)	−0.04	−0.24, 0.16	0.7
Race			
African American	—	—	
Asian	−0.41	−1.6, 0.76	0.5
Bi-Racial	0.29	−0.28, 0.86	0.3
Caucasian	0.15	−0.02, 0.32	0.075
Hispanic	−0.3	−0.96, 0.35	0.4
Native American	0.09	−1.0, 1.2	0.9
Other	0.78	−0.35, 1.9	0.2
Sex			
Female	—	—	
Male	−0.22	−0.39, −0.04	**0.015**
Age (per 5 years)	0.02	0.00, 0.05	0.081
**IP-10 (log)**			
CD4 Absolute (per 1000)	−0.57	−0.80, −0.34	**<0.001**
Race			
African American	—	—	
Asian	−0.1	−1.4, 1.2	0.9
Bi-Racial	0.19	−0.46, 0.84	0.6
Caucasian	0.13	−0.07, 0.34	0.2
Hispanic	−0.29	−1.2, 0.65	0.5
Native American	0.2	−1.1, 1.5	0.8
Other	0.21	−1.1, 1.5	0.7
Sex			
Female	—	—	
Male	−0.36	−0.57, −0.15	**<0.001**
Age (per 5 years)	0.03	−0.01, 0.06	0.15
**ICAM (log)**			
CD4 Absolute (non-linear)	1.56	—	0.2
Race			
African American	—	—	
Asian	0.63	−0.37, 1.6	0.2
Bi-Racial	0.32	−0.17, 0.82	0.2
Caucasian	0.4	0.24, 0.56	**<0.001**
Hispanic	0.56	−0.15, 1.3	0.12
Native American	0.26	−0.71, 1.2	0.6
Other	−0.1	−1.1, 0.88	0.8
Sex			
Female	—	—	
Male	−0.24	−0.41, −0.08	**0.004**
Age (non-linear)	1.38	—	0.4
**VCAM (log)**			
CD4 Absolute (per 1000)	−0.28	−0.38, −0.17	**<0.001**
Race			
African American	—	—	
Asian	0.38	−0.24, 1.0	0.2
Bi-Racial	−0.15	−0.45, 0.16	0.4
Caucasian	0.2	0.10, 0.29	**<0.001**
Hispanic	0.27	−0.09, 0.62	0.14
Native American	−0.5	−1.1, 0.10	0.1
Other	0.42	−0.19, 1.0	0.2
Sex			
Female	—	—	
Male	0.03	−0.07, 0.12	0.6
Age (non-linear)	1.91	—	0.078
**sCD14 (log)**			
CD4 Absolute (non-linear)	1.83	—	0.11
Race			
African American	—	—	
Asian	−0.18	−0.74, 0.38	0.5
Bi-Racial	−0.06	−0.34, 0.22	0.7
Caucasian	0.11	0.03, 0.19	**0.009**
Hispanic	0.23	−0.09, 0.54	0.2
Native American	0.13	−0.42, 0.67	0.7
Other	0.07	−0.48, 0.62	0.8
Sex			
Female	—	—	
Male	−0.1	−0.18, −0.01	**0.025**
Age (non-linear)	1.64	—	**0.04**
**sCD163 (log)**			
CD4 Absolute (per 1000)	−0.12	−0.28, 0.04	0.15
Race			
African American	—	—	
Asian	0.16	−0.80, 1.1	0.7
Bi-Racial	0.12	−0.35, 0.60	0.6
Caucasian	0.11	−0.03, 0.25	0.14
Hispanic	0.08	−0.47, 0.63	0.8
Native American	−0.34	−1.3, 0.60	0.5
Other	0.29	−0.66, 1.2	0.5
Sex			
Female	—	—	
Male	−0.12	−0.27, 0.03	0.11
Age (non-linear)	1.84	—	**0.006**
**D-dimer (log)**			
CD4 Absolute (per 1000)	−0.42	−0.79, −0.06	**0.022**
Race			
African American	—	—	
Asian	−0.39	−2.5, 1.7	0.7
Bi-Racial	−0.11	−1.2, 0.94	0.8
Caucasian	−0.3	−0.61, 0.01	0.057
Hispanic	−0.11	−1.3, 1.1	0.9
Native American	−2.4	−4.5, −0.35	**0.022**
Other	−0.85	−2.9, 1.2	0.4
Sex			
Female	—	—	
Male	−0.18	−0.51, 0.14	0.3
Age (non-linear)	1.64	—	0.2

^1^ For linear terms, estimates represent regression coefficients (β). For non-linear terms, estimates represent estimated degrees of freedom (EDF). IL-6 = Interleukin-6; VCAM = Vascular Cell Adhesion Molecule-1; ICAM = Intercellular Adhesion Molecule-1; TNF-RI = Tumor Necrosis Factor Receptor-1; TNF-RII = Soluble Tumor Necrosis Factor Receptor-2; hsCRP = High-sensitivity C-reactive protein; IP-10: Interferon-gamma-induced protein 10; OxLDL = Oxidized Low-Density Lipoprotein; sCD14 = Soluble CD14; sCD163 = Soluble CD163; IFABP = Intestinal fatty-acid binding protein; LBP = Lipopolysaccharide binding protein; BDG: Beta-D-glucan. Statistically significant *p*-values (*p* < 0.05) are shown in bold.

**Table A3:** Separate generalized additive models (GAMs) examining associations between cardiometabolic biomarkers and the covariates of absolute CD4 count (per 1000 units), race, sex, and age as covariates. Models also controlled %NOVA 1 and %NOVA 4 in diet.

Cardiometabolic Biomarker	Estimate: Beta/EDF ^1^	95% CI	*p*-Value
**Triglycerides (log)**			
CD4 Absolute (non-linear)	1.44	—	**0.001**
Race			
African American	—	—	
Asian	−0.74	−1.8, 0.35	0.2
Bi-Racial	0.33	−0.21, 0.88	0.2
Caucasian	0.23	0.08, 0.39	**0.004**
Hispanic	0.16	−0.46, 0.78	0.6
Native American	1.3	0.28, 2.4	**0.014**
Other	0.27	−0.80, 1.3	0.6
Sex			
Female	—	—	
Male	0.03	−0.14, 0.19	0.7
Age (per 5 years)	0.05	0.02, 0.07	**<0.001**
**Cholesterol (log)**			
CD4 Absolute (per 1000)	0.1	0.03, 0.18	**0.007**
Race			
African American	—	—	
Asian	−0.03	−0.47, 0.41	>0.9
Bi-Racial	0.09	−0.12, 0.31	0.4
Caucasian	0	−0.06, 0.07	>0.9
Hispanic	−0.07	−0.321, 0.19	0.6
Native American	0.35	−0.09, 0.77	0.11
Other	−0.25	−0.68, 0.18	0.3
Sex			
Female	—	—	
Male	−0.06	−0.13, 0.00	0.07
Age (per 5 years)	0.01	0.00, 0.02	0.2
**non-HDL**			
CD4 Absolute (per 1000)	22	9.2, 34	**<0.001**
Race			
African American	—	—	
Asian	−14	−87, 59	0.7
Bi-Racial	31	−4.8, 68	0.089
Caucasian	7	−3.6, 18	0.2
Hispanic	−18	−59, 24	0.4
Native American	72	0.67, 143	**0.048**
Other	−30	−102, 42	0.4
Sex			
Female	—	—	
Male	−5.9	−17, 5.2	0.3
Age	0.21	−0.14, 0.56	0.2
**LDL**			
CD4 Absolute (non-linear)	1.62	—	**0.046**
Race			
African American	—	—	
Asian	0.73	−64, 65	>0.9
Bi-Racial	22	−9.9, 54	0.2
Caucasian	1.2	−8.2, 11	0.8
Hispanic	−13	−50, 23	0.5
Native American	30	−33, 93	0.3
Other	−31	−95, 32	0.3
Sex			
Female	—	—	
Male	−6.7	−17, 3.1	0.2
Age (per 5 years)	0.02	−1.5, 1.6	>0.9
**Glucose (log)**			
CD4 Absolute (non-linear)	1.37	—	0.059
Race			
African American	—	—	
Asian	−0.07	−0.42, 0.27	0.7
Bi-Racial	−0.04	−0.21, 0.13	0.6
Caucasian	0.04	−0.01, 0.09	0.12
Hispanic	−0.09	−0.28, 0.11	0.4
Native American	0.14	−0.19, 0.48	0.4
Other	0.21	−0.13, 0.55	0.2
Sex			
Female	—	—	
Male	−0.04	−0.09, 0.02	0.2
Age (per 5 years)	0.01	0.00, 0.01	0.2
**HOMA-IR (log)**			
CD4 Absolute (per 1000)	0.24	0.04, 0.45	**0.018**
Race			
African American	—	—	
Asian	−0.54	−1.7, 0.66	0.4
Bi-Racial	0.04	−0.55, 0.62	>0.9
Caucasian	0.11	−0.06, 0.28	0.2
Hispanic	−0.15	−0.82, 0.52	0.7
Native American	0.69	−0.47, 1.8	0.2
Other	0.82	−0.34, 2.0	0.2
Sex			
Female	—	—	
Male	−0.24	−0.42, −0.06	**0.01**
Age (per 5 years)	0.01	−0.02, 0.04	0.4
**RHI**			
CD4 Absolute (per 1000)	0.03	−0.17, 0.22	0.8
Race			
African American	—	—	
Asian	0.47	−0.64, 1.6	0.4
Bi-Racial	0.75	0.20, 1.3	**0.008**
Caucasian	0.19	0.03, 0.35	**0.023**
Hispanic	−0.29	−0.92, 0.34	0.4
Native American	−0.6	−1.7, 0.48	0.3
Other	−0.5	−1.6, 0.59	0.4
Sex			
Female	—	—	
Male	0.04	−0.13, 0.22	0.6
Age (per 5 years)	−0.02	−0.04, 0.01	0.2
**Systolic BP**			
CD4 Absolute (per 1000)	−4.4	−9.6, 0.90	0.1
Race			
African American	—	—	
Asian	17	−13, 48	0.3
Bi-Racial	−7.7	−23, 7.5	0.3
Caucasian	−4.1	−8.6, 0.34	0.07
Hispanic	−14	−31, 3.2	0.11
Native American	−2.7	−33, 27	0.9
Other	−12	−42, 18	0.4
Sex			
Female	—	—	
Male	0.25	−4.4, 4.9	>0.9
Age (non-linear)	2.62	—	**0.041**
**Diastolic BP**			
CD4 Absolute (per 1000)	−3.8	−7.1, −0.60	0.02
Race			
African American	—	—	
Asian	−4.2	−23, 15	0.7
Bi-Racial	−1.4	−11, 8.0	0.8
Caucasian	−3.6	−6.4, −0.81	**0.012**
Hispanic	−5.3	−16, 5.5	0.3
Native American	−0.99	−20, 18	>0.9
Other	−23	−42, −4.7	**0.014**
Sex			
Female	—	—	
Male	−1.8	−4.7, 1.1	0.2
Age	0.07	−0.03, 0.16	0.2

^1^ For linear terms, estimates represent regression coefficients (β). For non-linear terms, estimates represent estimated degrees of freedom (EDF). BP: Blood Pressure; HOMA-IR: Homeostatic Model Assessment of Insulin Resistance; LDL = Low-Density Lipoprotein; HDL = High-Density Lipoprotein; RHI: Reactive Hyperemic Index. Statistically significant *p*-values (*p* < 0.05) are shown in bold.
